# Target delivery of MYCN siRNA by folate-nanoliposomes delivery system in a metastatic neuroblastoma model

**DOI:** 10.1186/1475-2867-13-65

**Published:** 2013-06-27

**Authors:** Qiqi Zhu, Chen Feng, Weiwei Liao, Yan Zhang, Suoqin Tang

**Affiliations:** 1 Department of Pediatrics, Hainan Brach of Chinese PLA General Hospital, Haitang Bay, Sanya, Hainan Province 572013, China

**Keywords:** Folate-nanoliposomes, Metastatic neuroblastoma, MYCN, siRNA

## Abstract

**Background:**

Folate-nanoliposomes delivery system has emerged recently as a specific and safety delivery method and gradually used as the carrier of a variety kinds of drugs including compounds, plasmids and siRNAs.

**Methods:**

In this study, we established a bone marrow and bone metastasis xenograft mouse model by injecting the LA-N-5 cell into the bone marrow cavity. Fluorescence microscopy, TUNEL Assay, Quantitative RT-PCR and western blot were conducted to analysis the distribution of folate-nanoliposomes entrapped MYCN (V-myc myelocytomatosis viral related oncogene) siRNA in mice and the relevant suppression effect.

**Results:**

The folate-nanoliposomes entrapped MYCN siRNA can be specifically distributed in tumor tissues. Further study shows that folate-nanoliposomes entrapped MYCN siRNA lead to MYCN mRNA expression significantly down-regulated (>50%, and p < 0.05) compared with negative control siRNA treatment. MYCN protein expression was inhibited about 60% in vivo, thus induced tumor cell apoptosis markedly.

**Conclusion:**

This study point to a new way for treatment of metastatic neuroblastoma and could widen the application of folate-nanoliposomes delivery system in tumor therapy.

## Introduction

Neuroblastoma is the most common extracranial solid tumor occurring in children, which accounts for about 15% of all childhood cancer deaths [1,2]. It is generally accepted that the amplification of the MYCN proto-oncogene is highly relevant to the genesis and prognosis of neuroblastoma [3,4]. MYCN is an important member of the myc family that includes 3 strong oncogenes, MYC, MYCN, and MYCL [5]. Targeting MYCN gene has developed into a promising therapeutic strategy [6-8]. Small interference RNA (siRNA) is synthetic short double-stranded RNA, which can induce the destruction of homologous mRNA when introduced into a cell [9]. siRNA has been developed into an effective tool for suppressing target proteins expression by specifically digesting their mRNAs [10]. siRNAs have been widely investigated as therapeutic agents to treat a wide range of human diseases including neuroblastoma [7,11].

Folate, a nontoxic low-weigh compound, is vital for tumor cell proliferation and survival. Studies show that tumor cells can express 200-folds more folate receptor on the membrane than normal cells, which is accordance to the high intake of folate in tumor cells. Folate receptor is overexpressed in a wide range of human cancer cells, including ovarian cancer, endometrial cancer, head and neck cancers, myeloid leukaemia, and some pediatric cancers [12]. Folate conjugating on liposomes with entrapped drugs [13-16] or plasmids [17-20] has been used successfully to deliver drugs to folate-receptor-expressing cancer cells. Our previous study had revealed that the folate receptor was highly expressed on the membrane of LA-N-5 neuroblastoma cells. In this study, we want to further investigate whether folate receptor-targeted liposome can act as a good delivery tool of MYCN siRNA and exert a satisfying therapeutic effect on neuroblastoma.

In this study, we established a bone marrow and bone metastasis xenograft mouse model by injecting the LA-N-5 cell into the bone marrow cavity. Then we examined whether folate-nanoliposomes entrapped MYCN siRNA can specifically distribute to tumor tissues and suppress MYCN gene expression as well as induce apoptosis in neuroblastoma cells.

## Materials and methods

### Animals

BALB/c nude mice (4 wk) were used for all experiments. All animals were housed in the Experimental Animal Centre of PLA General Hospital, and all procedures were approved by the Institutional Animal Care and Use Committee of PLA General Hospital.

### Cell culture

LA-N-5 neuroblastoma cell was the generous gift of Dr. Robert Seeger from Children’s Hospital Los Angeles, and the cells were grown in RPMI 1640 medium (GIBCO BRL, USA) containing 15% fetal bovine serum (Hyclone, USA), 2 mmol/L l-glutamine, 100 IU/ml penicillin G and 100 μg/mL streptomycin (all were from Huamei Company, China) in a humidified 5% CO_2_ and 95% air atmosphere at 37°C.

### Establishment of the bone marrow and bone metastasis xenograft mouse model

Previously described procedure was introduced in the current study [21,22]. Briefly, LA-N-5 neuroblastoma cells were harvested and washed by centrifugation and resuspended in cold serum-free medium prior to injection. Mice were immobilized in a restraining device, and cells (10^5^ cells in 5 μl) were injected into the bone marrow cavity of the femoral proximal metaphysis. About five weeks later, the diameters of tumors can be up to 5 mm and the bone metastasis model is successfully established.

### Preparation of the folate-nanoliposome entrapped MYCN siRNA

The folate-nanoliposome entrapped MYCN siRNA with Cy-3 fluorescence tag was the generous gift from Dr. Robert J. Lee (Ohio State University, College of Pharmacy). siRNA sequences were as follows: MYCN siRNA sense: 5′-CGGAGAUGCUGCUUGAGAA dtdt-3′, anti-sense: 5′-UUCUCAAGCAGCAUCUCCG dtdt-3′; the negative control siRNA: sense: 5′-UUCUCCGAACGUGUACGU dtdt-3′, anti-sense: 5′-ACGUGACAC GUUCGGAGAA dtdt-3′.

### siRNA distribution analysis and gene therapy

For siRNA distribution analysis, LA-N-5 neuroblastoma cells were injected into mice bone marrow cavity to establish the bone marrow and bone metastasis xenograft models. Then the folate-nanoliposome entrapped MYCN siRNA with Cy3 tag (3 mg/kg) was injected into mice via lateral tail vein, and mice were sacrificed 8 hours after injection. Tumors of femur and other indicated organs (heart, lung, liver, kidney) were harvested, embedded in optimum cutting temperature compound (OCT) and sectioned into 4 μm frozen sections. Tissues sections were observed under fluorescence microscopy (stimulating wavelength, 554 nm). Three views of each organ were chosen randomly and subjected to calculate the average integrated optical density (IOD) with Image pro plus 6.0 Software. In the gene therapy experiment, mice were divided into two groups (6 mice for each group). LA-N-5 neuroblastoma cells were injected into mice bone marrow cavity to establish the bone marrow and bone metastasis xenograft model. Then the folate-nanoliposome entrapped MYCN siRNA with Cy3 tag (3 mg/kg/d) were injected into one group of mice by lateral tail vein. The other group of mice were injected with folate-nanoliposome entrapped the control siRNA (3 mg/kg/d). Mice were sacrificed at the sixth day after 5 injections of folate-nanoliposome entrapped siRNAs. Tumors frozen sections were analyzed by TUNEL Assay.

### Quantitative RT-PCR analysis

The cells were harvested and RNA was extracted by using the Ultrapure RNA Kit (CWbio.Co.Ltd). Briefly, cells were lysed in RLT buffer and RNA was purified according to the manufacturer’s instructions. RNase-free DNase set (CWbio.Co.Ltd) was used to remove any contaminating genomic DNA. The cDNA was synthesized using HiFi-MMLV cDNA Kit (CWbio.Co.Ltd). The primer sequences for MYCN were: 5′-CTCAGTACCTCCGGAGAG-3′ (sense) and 5′-GGCATCGTIGAGGATC-3′ (antisense). The primer sequences for GAPDH were:5′-TGCACCACCAACTGCTTAGC-3′ (sense) and 5′-GGCATGGACTGTGGTCATGAG-3′ (antisense). Real-time PCR was performed on an Roche LightCycler 480IIsystem, using LightCycler 480 SYBR GreenIMaster (Roche). Each sample was determined in triplicate. MYCN mRNA expression was calculated as MYCN copies/GAPDH copies. Relative standard curve method was used to determine the relative mRNA expression of MYCN gene.

### Western blot

Tumor frozen samples from each mouse in a same group were mixed and total protein was extracted. Proteins were separated by a precast 4-12% SDS-PAGE gel (Sigma) and electrophoretically transferred to a PVDF membrane, followed by incubation with anti-β-actin antibody (1/2000 ab3280 Abcam, Cambridge, MA, USA) and anti-n-Myc antibody [NCM II 100] (1:1000 ab16898 Abcam, Cambridge, MA, USA). Protein bands were detected with ECL reagents (Amersham Biosciences, Buchinghamshire, UK) and visualized by autoradiography.

### TUNEL assay

Briefly, frozen tumor samples were cut on a cryostat and got 4 μm-thick sections. Slices were fixed with acetone and soaked in 3% hydrogen peroxide-methanol solution for 20 min, then rinsed in PBS for 3 times. Fixed sections were incubated with proteinase K (20 μg/ml) for 20 min at 37°C and washed with TBS for 3 times. Then they were incubated with TdT buffer containing digoxigenin labelled desoxyuridinetriphosphate (DIG-UTP) and dATP for 2 h at 37°C, and immersed in stop solution for 30 min at room temperature. After washing, the sections were incubated with strept avidin-biotin-peroxidase complex (SABC) antibody solution for 60 min at 37°C, followed by incubation with DAB for 20 min. Slices were counterstained with hematoxylin and examined with a fluorescence microscope.

### Statistical analysis

All experiments were performed at least in triplicate and data are expressed as means ± SEM. Statistical differences were analyzed by two-tailed *t* test. P < 0.05 was considered statistically significant.

## Results

### Folate-nanoliposome entrapped MYCN siRNA is specifically distributed in tumor tissues

**Figure 1:**
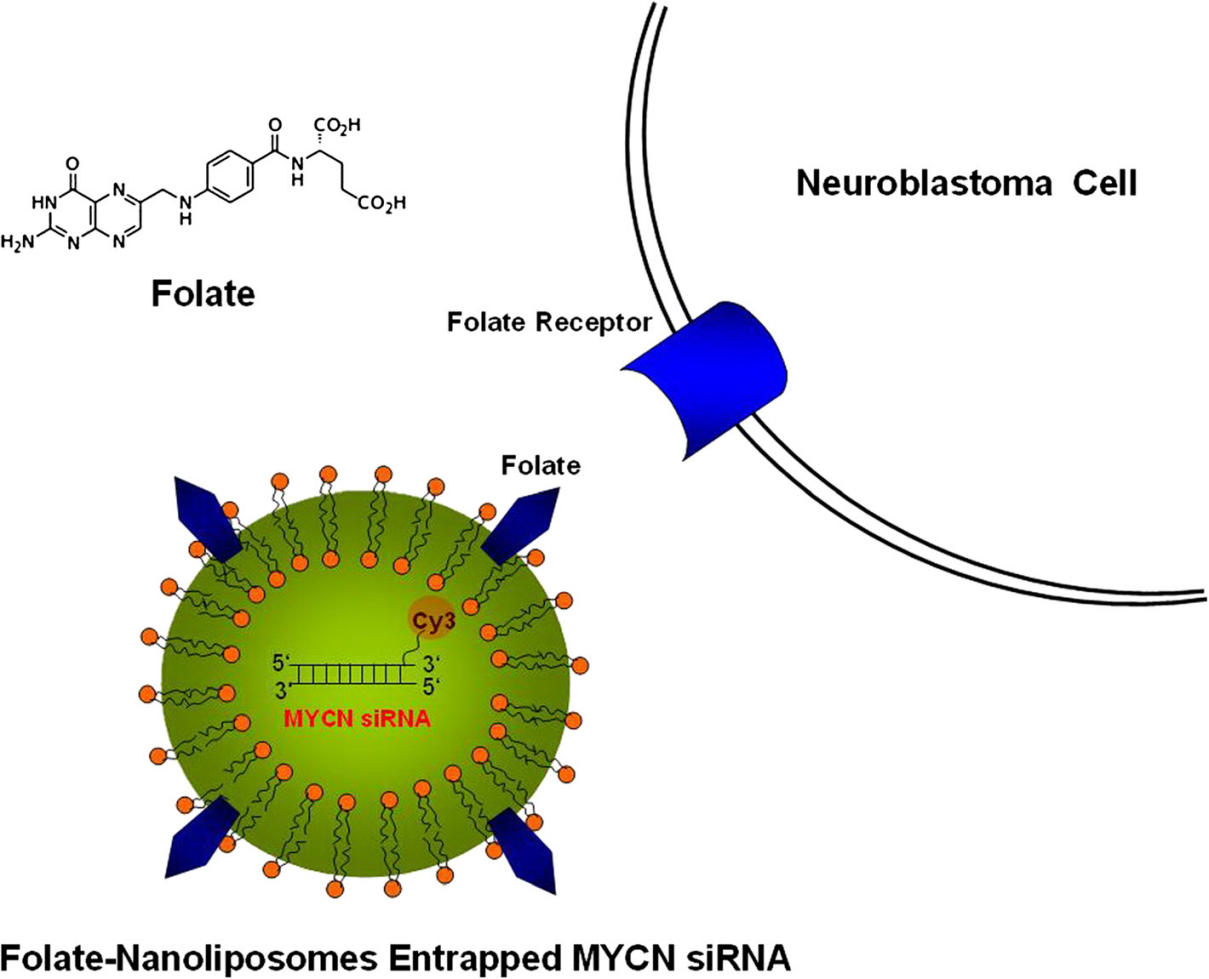
A diagram of target delivery of MYCN siRNA by folate-nanoliposomes.

**Figure 2:**
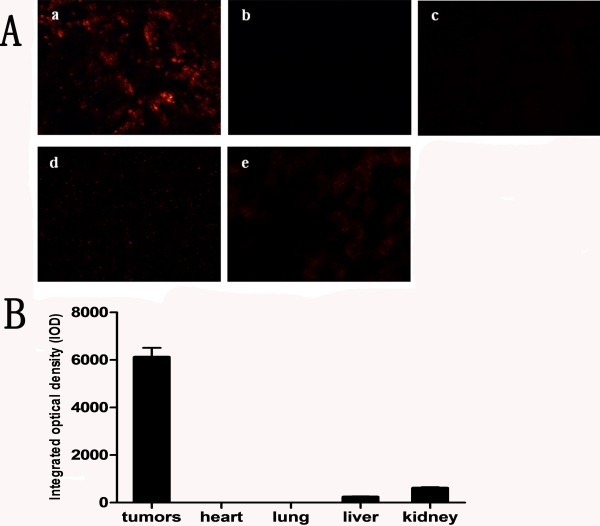
**Distribution of folate-nanoliposome entrapped MYCN siRNA: A, Cy3 fluorescence of different tissues. a**, tumors tissue; **b**, heart; **c**, lung; **d**, liver; **e**, kidney. This graph is one representative result of three views; **B**, Quantitative analysis of fluorescence intensity of each tissue. Fluorescence intensity was expressed as integrated optical density (IOD). Results are expressed as means ± SE.

LA-N-5 neuroblastoma cells were injected into mice bone marrow cavity to establish the bone marrow and bone metastasis xenograft model. Then mice were injected with the folate-nanoliposome entrapped MYCN siRNA with Cy3 tag (Figure 1) by tail vein, and sacrificed 8 hours after injection. Tissues sections were observed under fluorescence microscopy and showed that Cy3 fluorescence was very bright in tumors in bones (Figure 2A, B). Liver, kidney, heart and lung tissues have weak or no visible fluorescence (Figure 2A, B). This indicates that folate-nanoliposome entrapped MYCN siRNA is specifically distributed in tumor tissues.

### In vivo gene silencing effect of folate-nanoliposome entrapped MYCN siRNA in LA-N-5 neuroblastoma

**Figure 3:**
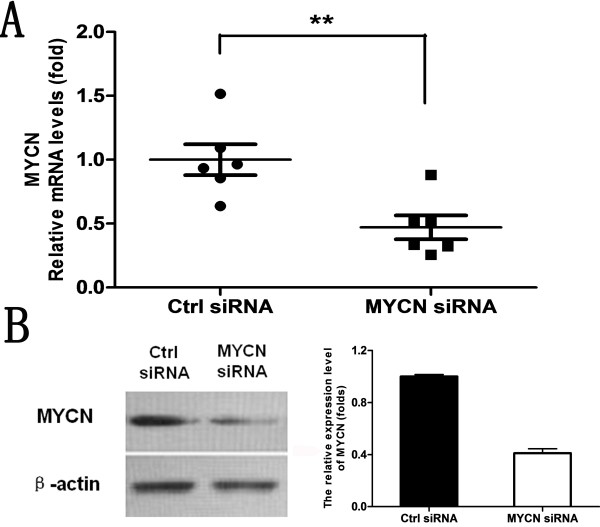
**The in vivo gene silencing effect of folate-nanoliposome entrapped MYCN siRNA in LA-N-5 neuroblastoma: A, Quantitative RT-PCR analysis of MYCN gene expression.** Results are normalized to GAPDH expression and are presented as fold increases over the control. 6 mice each group, *P < 0.05, **P < 0.01; **B**, Western blot analysis of MYCN gene expression. Frozen tumor samples of each mouse in a same group were mixed and analyzed by Western blot. The right graph is a quantitative analysis of the left one.

Mice were injected (i.v.) with folate-nanoliposome entrapped MYCN siRNA or control siRNA for 5 days and gene silencing effect of MYCN siRNA in LA-N-5 neuroblastoma were examined by real-time PCR and Western blot. As shown in Figure 3A, MYCN mRNA expression was significant down-regulated (>50%) by folate-nanoliposome entrapped MYCN siRNA compared to negative control siRNA treatment (p < 0.01). Western blot result showed MYCN protein expression was about 60% inhibited by folate-nanoliposome entrapped MYCN siRNA (Figure 3B). These data demonstrate that systematic injection of folate-nanoliposome entrapped MYCN siRNA can silence MYCN gene expression of tumor tissues efficiently.

### The pro-apoptotic effect of folate-nanoliposome entrapped MYCN siRNA in neuroblastoma

**Figure 4:**
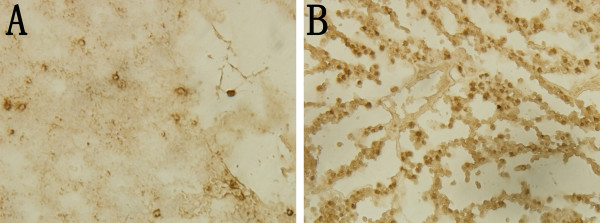
TUNEL assay of LA-N-5 neuroblastoma: A, folate-nanoliposome entrapped control siRNA group; B, folate-nanoliposome entrapped MYCN siRNA group.

Gene therapeutic effect of folate-nanoliposome entrapped MYCN siRNA was evaluated by TUNEL assay of neuroblastoma. As shown in Figure 4, MYCN siRNA treatment group caused much more TUNEL positive cells than control siRNA treatment group. This indicates that folate-nanoliposome entrapped MYCN siRNA treatment can lead to apoptosis of neuroblastoma in vivo.

## Discussion

Our results showed MYCN knockdown induces apoptosis of metastatic neuroblastoma in vivo, MYCN gene, located on the short arm of chromosome 2, is only expressed during the stages of embryonic development of the nervous system, kidney, lung and spleen [23]. MYCN gene is considered a proto-oncogene associated with the malignant tumors growth [24-26]. Since MYCN gene amplification plays a key role in the neuroblastoma progression, inhibition the MYCN gene expression becomes a probable strategy for high-risk neuroblastoma therapy. Studies by other groups [27-29] have demonstrated that inhibition of MYCN gene expression in neuroblastoma cells makes cell cycle arrest at G1 phase, and promotes tumor cell apoptosis or differentiation into neural cells. The MYCN gene is a promising drug target for treating peripheral and central nervous system tumors [30].

Although the therapeutic strategy targeting MYCN in vivo by siRNA is attractive, the traditional drugs delivery methods seem to be nonspecific and toxic [31]. The folate receptor-targeted liposome delivery system has emerged recently as a specific and safety delivery method and become the frontier of drugs delivery. This study was designed to examine the specificity and efficacy of folate-nanoliposome entrapped MYCN siRNA in treatment of metastatic neuroblastoma, showing that MYCN siRNA was distributed specically in tumor tissues under the direction of folate-folate receptor interaction and further silences MYCN gene expression both in mRNA and protein levels and finally induces apoptosis of neuroblastoma cells. This indicates that folate-nanoliposome entrapped MYCN siRNA could be used in future gene therapy of neuroblastoma. Of note, the in vivo gene knockdown efficiency is up to 60% in protein level, which is much higher than that of traditional methods such as lipofection or calcium-phosphate transfection [31].

This study points to a new way for treatment of metastatic neuroblastoma and widen the application of folate-nanoliposomes delivery system in tumor therapy. The high specificity, efficiency and safety make folate receptor-targeted liposome delivery system a killer mace in siRNAs-mediated cancer gene therapy.

## Abbreviations

siRNA: Small interference RNA; IOD: Integrated optical density; DIG-UTP: Digoxigenin labelled desoxyuridinetriphosphate; SABC: Strept avidin-biotin-peroxidase complex.

## Competing interests

The authors declare that they have no competing interests.

## Authors’ contributions

The work presented here was carried out in collaboration between all authors. ST defined the research theme. QZ designed methods and experiments, carried out the laboratory experiments, CF and WL analyzed the data, interpreted the results. YZ co-worked on associated data collection and their interpretation. All authors read and approved the final manuscript.
